# Bacterial toxin activation and deployment: Context-dependent links to host immune remodeling

**DOI:** 10.1080/21505594.2026.2753301

**Published:** 2026-10-29

**Authors:** Hao Xu, Chao Li, Xinchao Yi, Cheng Huang, Jinmei Zhang, Yuan Li, Ying Huang, Jing Chen, Jie Lu, Bin Liu, Jun He

**Affiliations:** a Department of Clinical Laboratory, Affiliated Nanhua Hospital, Hengyang Medical College, University of South China, Hengyang, China; b Department of Clinical Laboratory, the Second Affiliated Hospital, Hengyang Medical College, University of South China, Hengyang, China; c Department of Blood Transfusion, the Second Affiliated Hospital, Hengyang Medical College, University of South China, Hengyang, China; d School of Basic Medical Sciences, Youjiang Medical University for Nationalities, Baise, Guangxi, China

**Keywords:** Bacterial toxins, activation, immune remodeling, host–pathogen interactions, virulence mechanisms, cell death

## Abstract

Many bacterial toxins are produced in latent or structurally constrained forms and acquire functional competence or productive access to host targets through distinct activation and deployment processes. This review distinguishes protein-level activation – including proteolytic maturation, reductive subunit release, metabolite-triggered autoprocessing, and host-factor-dependent catalytic activation – from receptor-mediated uptake, intracellular trafficking and translocation, vesicle-mediated delivery, and bacterial or phage-associated control of toxin expression and release. Across representative systems, these processes govern effector competence, availability, and localization and are associated with three broad immune outcomes: disruption of inflammatory signaling and cytokine networks, impairment of humoral defenses, and cell injury or regulated cell death. Their immune effects are often indirect and vary with host niche, cell type, receptor repertoire, trafficking route, and exposure timing. Distinguishing direct, effector-mediated, injury-mediated, availability-mediated, and unresolved relationships clarifies the boundaries of toxin activation and identifies priorities for in vivo validation and anti-virulence intervention.

## Introduction

Bacterial toxins are pivotal determinants of pathogenesis, yet many are synthesized, secreted, or delivered in latent or structurally constrained forms that require defined molecular processing before becoming functionally competent. This “delayed activation” strategy can prevent premature toxin activity during bacterial production and extracellular transit while enabling functional maturation after the toxin encounters appropriate host-associated conditions [1]. However, molecular activation should be distinguished from subsequent processes such as intracellular trafficking, membrane translocation, vesicle-mediated delivery, and regulated toxin expression or release, which control toxin availability and deployment without necessarily activating the catalytic domain itself.

Current molecular evidence supports several distinct mechanisms of toxin activation and deployment. Host protease-mediated licensing, exemplified by furin-dependent processing, can convert precursor toxins into primed or functionally competent forms [1]. Host-cell physicochemical cues – including redox conditions and metabolite ligands such as inositol hexakisphosphate (InsP_6_) – can promote subunit dissociation or intrinsic autoprocessing in specific intracellular compartments [2–4]. By contrast, endosomal acidification may primarily drive conformational rearrangement, membrane insertion, and translocation, as occurs for botulinum neurotoxin and diphtheria toxin, rather than directly activating their catalytic domains. At the bacterial-population level, prophage induction and lysis-associated secretion can regulate toxin expression, release timing, and local exposure dose, but these processes represent deployment regulation rather than protein-level activation [5–9].

Once molecularly processed and delivered to their sites of action, toxin effectors can alter host immunity through disruption of signaling pathways, remodeling of cytokine responses, dismantling of humoral defenses, and induction of regulated cell death [10–16]. Nevertheless, the resulting immune phenotypes do not arise through a single mechanistic route. In some systems, activated toxin effectors directly target immune signaling components; in others, immune alterations occur indirectly through epithelial barrier disruption, cellular stress, tissue injury, or the loss of immune cells. Activation should therefore not be assumed to determine immune outcomes uniformly without considering toxin architecture, host-cell context, effector location, and the strength of the available mechanistic evidence.

In this review, we use “protein-level toxin activation” in a restricted mechanistic sense to describe molecular events that convert latent or structurally constrained toxin forms into functionally competent states. We distinguish these events from receptor-mediated uptake, intracellular trafficking and translocation, vesicle-mediated delivery, and bacterial or phage-associated regulation of toxin expression and release, which instead govern effector localization and availability. We organize representative toxin systems according to these mechanistic levels and evaluate whether their relationships to immune remodeling are direct, effector-mediated, injury-mediated, availability-mediated, or unresolved.

## Activation pathways of bacterial protoxins

Bacterial toxins achieve functional competence through several mechanistically distinct processes that operate at different stages of intoxication (Table 1). In this review, protein-level activation refers specifically to molecular events that convert latent or structurally constrained toxin precursors into functionally competent states, including proteolytic maturation, reductive subunit dissociation, metabolite-triggered autoprocessing, and host-factor-dependent catalytic activation. By contrast, receptor-mediated uptake, intracellular trafficking, membrane translocation, and bacterial regulation of toxin expression or release determine toxin availability, localization, and effector delivery without necessarily activating the catalytic domain itself. This distinction is important because the site at which a toxin is processed, the route by which it is transported, and the site at which its effector acts are often spatially and mechanistically distinct. Moreover, downstream inflammatory or immunological phenotypes may arise indirectly from cytotoxicity, barrier disruption, membrane injury, or cell death rather than from the activation event itself. Table 1 therefore distinguishes these mechanistic layers and evaluates the nature of their linkage to reported immune outcomes.

**Table 1.** Evidence-based comparison of bacterial toxin protein-level activation, deployment, expression/release regulation, and associated immune outcomes.

Toxin system	Activation, deployment, or regulatory trigger	Site of activation, deployment, or regulation	Translocation/ deployment route	Effector site	Immune outcome	Evidence level / nature of linkage to immune outcome
**A. Protein-level activation and processing**						
*Pseudomonas aeruginosa* exotoxin A (PE)	Furin cleavage exposes a translocation-competent toxin [17,19]	TGN/endosome	Endocytosis → cytosol	Cytosolic translation machinery	Translation arrest; NLRP1-dependent epithelial injury in selected models [105]	Indirect – effector-mediated; activation-to-immune linkage not directly isolated
*Aeromonas hydrophila* proaerolysin	Furin-mediated cell-surface processing; PACE4/PC5/6A also cleave in vitro [20]	Cell surface/extracellular milieu	Membrane binding → pore insertion	Plasma membrane	Cell lysis; secondary inflammation	Indirect – injury-mediated
*Clostridium perfringens* enterotoxin (CPE)	Trypsin removes N-terminal residues and enhances pre-existing activity [21,22]	Intestinal lumen	Claudin binding → membrane pore	Epithelial membrane/tight junctions	Barrier loss and permeability; secondary inflammation	Indirect – injury-mediated
Anthrax toxin (protective antigen, lethal factor, and edema factor; PA/LF/EF)	PA83→PA63 furin cleavage; low-pH pore conversion [111–114]	Cell surface → endosome	Endocytosis → PA pore → cytosol	Cytosolic LF/EF	Inflammasome/pyroptosis and immune modulation [106,107,111,116]	Indirect – effector-mediated
Botulinum neurotoxin (BoNT)	Disulfide reduction releases LC [27,30–34]	Cytosolic face of the synaptic endosome	Neuronal receptor binding and endocytosis → low-pH HN insertion → LC translocation	Cytosolic SNAREs	Neurotransmission blockade with context-dependent neuroimmune effects	Indirect – effector-mediated
Diphtheria toxin (DT)	Furin cleavage generates a primed A–B intermediate [23–25]	Cell surface/endosome	Receptor uptake → A-fragment cytosolic delivery	Cytosolic eEF-2	Translation arrest, apoptosis, and immune-cell loss	Indirect – effector-mediated
Cholera toxin (CT)	CTA nicking by bacterial proteases or host serine proteases → ER reduction/PDI-assisted CTA1 release → ARF-dependent activation [2,37,38,40]	Before/during entry (nicking); ER (CTA1 release); cytosol (ARF-dependent activation)	Endosome → Golgi → ER → ERAD-like cytosolic export	Cytosolic Gsα	cAMP-dependent IL-12 suppression and altered DC differentiation [39]	Indirect – effector-mediated
Shiga toxin (Stx): protein processing	Furin-mediated A-chain nicking → ER disulfide reduction and StxA1 release [45–47]	Endocytic/retrograde compartments (nicking); ER (disulfide reduction)	Gb3 uptake → Golgi/ER trafficking → ERAD-like retrotranslocation	Cytosolic 28S rRNA[50]	Ribotoxic injury and context-dependent inflammasome modulation [51,52]	Indirect – injury-mediated
Repeats-in-toxin proteins with a self-processing module (RTX-SPM; Asp-Pro subtype)	Ca^2+^-triggered SPM autoproteolysis [53,54]	Extracellular/cell-surface milieu	N-terminal effector release	Toxin-specific	Effector-specific cellular damage; immune consequences remain context-dependent	Unresolved
MARTX toxins: CPD-dependent effector release	Cytosolic InsP_6_ activates CPD autoprocessing and releases effector modules [64]	Host cytosol	Membrane translocation → CPD-mediated effector liberation	Effector-dependent: Rho/Rac, Ras/Rap1, and NAD(P)+-dependent pathways	Variant-dependent immune-gene suppression and modulation of ROS production; RDTND–RID suppresses ROS [86–88]	Indirect – effector-mediated; RID/RDTND immune effects and RRSP signaling demonstrated
*Vibrio vulnificus* MARTX MCF effector	After CPD-dependent release, host ARFs stimulate MCF autoprocessing and Rab-cleaving activity [63,65]	Host cytosol	CPD-mediated release → ARF-dependent MCF autoprocessing	C-terminal tails of Rab GTPases	Rab cleavage/degradation and organelle injury; specific immune consequences unresolved [65]	Unresolved for MCF-specific immune outcomes
*Photorhabdus luminescens* Makes caterpillars floppy 1 and 2 toxins (Mcf1/Mcf2)	ARF-dependent activation of Mcf1 autoproteolysis; Mcf2 is a related homolog with a less-defined activation mechanism [66,67]	Host cytosol	Cytosolic toxin delivery → ARF-dependent conformational activation/autoprocessing	Cytosolic effector fragments (targets unresolved)	Insecticidal activity; immune consequences in mammalian systems unclear	Unresolved
Cholesterol-dependent cytolysins (CDCs)	Cholesterol binding → oligomerization/pore conversion [68]	Host plasma membrane	Direct membrane insertion	Plasma membrane	Membrane damage, inflammation, and cell death	Indirect – injury-mediated
*Staphylococcus aureus* α-hemolysin (Hla)	ADAM10 receptor engagement followed by oligomerization and pore formation [70]	Host plasma membrane	Receptor binding → heptameric pore assembly	ADAM10/E-cadherin axis and plasma membrane pores	Barrier disruption, NLRP3 inflammasome activation, and inflammatory cell death [89,108,109]	Indirect – effector-mediated
Large clostridial glucosylating toxins: *Clostridioides difficile* toxins A/B and *Clostridium sordellii* lethal/hemorrhagic toxins (TcdA/TcdB/TcsL/TcsH)	InsP_6_-dependent CPD autoprocessing releases GTD [3,4]	Endosome/cytosolic interface	Receptor binding → endocytosis → membrane translocation → cytosolic GTD release	Cytosolic small GTPases; Rho/Ras specificity varies by toxin	Barrier injury, inflammasome responses, and cell death [55–62]	Indirect – effector-mediated; some TcdB inflammasome responses are GTD-independent [57,61]
**B. Uptake, trafficking, translocation, and vesicle-mediated deployment**						
*Mycoplasma pneumoniae* community-acquired respiratory distress syndrome toxin (CARDS toxin)	Endosomal acidification-associated conformational remodeling [35,36]	Acidifying endosome	Annexin A2 uptake → KELED-dependent retrograde route	v-ATPase-associated vacuolar machinery	Vacuolization, epithelial injury, and inflammation	Indirect – injury-mediated
*Enterococcus* pore-forming toxins (Epxs; Epx2/Epx3)	HLA-I/MHC-I receptor recognition [69]	Host cell surface	HLA-I/MHC-I receptor binding → membrane targeting/pore formation	Host plasma membrane	Direct relationship to immune remodeling not established	Unresolved
Enterotoxigenic *Escherichia coli* heat-labile enterotoxin (ETEC LT)	OMV-associated packaging and vesicle-mediated delivery [75]	Bacterial envelope/OMV compartment	OMV-mediated extracellular delivery → intestinal epithelial uptake	Cytosolic Gsα signaling pathway	cAMP elevation, epithelial dysfunction, and inflammatory responses	Indirect – effector-mediated
Cytolethal distending toxin (CDT)	Vesicle-associated delivery and intracellular trafficking [76–78]	Extracellular vesicle environment	Endocytosis → retrograde trafficking → ER → NLS-dependent nuclear targeting	Nuclear CdtB DNase activity	DNA damage, cell-cycle arrest, and immune-cell dysfunction	Indirect – effector-mediated
*Salmonella enterica* serovar Typhi typhoid toxin	Host-derived vesicle carrier-mediated export [79]	Infected-cell vesicle compartment	Vesicle sorting → extracellular delivery → recipient-cell uptake	Nuclear/cytosolic toxin targets	Exposure-dependent cellular stress and downstream immune effects	Indirect – availability-mediated
**C. Expression and release regulation**						
*Staphylococcus aureus* Panton-Valentine leukocidin (PVL)	Prophage induction increases expression and lysis-linked release [5,6]	Bacterial prophage regulatory state	Bacterial lysis → extracellular toxin → leukocyte membrane	Leukocyte membrane	Neutrophil lysis and inflammatory injury	Indirect – availability-mediated
*Streptococcus pyogenes* pyrogenic exotoxin C (SpeC)	Prophage-linked expression/release [7]	Bacterial prophage regulatory state	Extracellular release → MHC II/TCR engagement	MHC II/TCR Vβ interface	T-cell activation and cytokine amplification [91–93]	Indirect – availability-mediated
*Corynebacterium diphtheriae* diphtheria toxin (DT) expression	Low iron relieves DtxR repression of *tox* [72,73]	Bacterial transcriptional regulatory state	Production/release → host uptake	After activation: cytosolic eEF-2	Dose-dependent translation arrest and tissue injury	Indirect – availability-mediated
Shiga toxin (Stx): phage-dependent release	RecA-SOS induction regulates *stx* expression, lytic release, and toxin availability [8,9]	Bacterial prophage regulatory state	Lytic pulse → Gb3 uptake → retrograde route	After ER processing: cytosolic ribosome	Exposure-dependent barrier injury and immune modulation [51,52]	Indirect – availability-mediated
*Yersinia entomophaga* toxin complex (YenTc)	Specialized subpopulation expression with T10SS-dependent controlled lysis program [68]	Bacterial population-level regulatory state	Toxin-producing minority cells → T10SS-dependent cell lysis → extracellular toxin deployment	Host target cells (after extracellular toxin delivery)	Host tissue damage/insecticidal activity	Indirect – availability-mediated

Note: Protein-level activation and processing denotes molecular events that convert latent or structurally constrained toxin precursors into functionally competent states. Deployment denotes receptor-mediated uptake, intracellular trafficking, membrane translocation, or vesicle-mediated delivery that governs toxin localization and effector access without necessarily altering the catalytic state of the toxin. Expression and release regulation denotes bacterial or phage-associated control of toxin abundance, timing, or availability. These three mechanistic layers are distinct but may occur sequentially within the same intoxication pathway. Linkage categories are defined as follows: “Direct” indicates that experimental perturbation of the specified event alters a defined immune phenotype and that a mechanistic link has been demonstrated; “Indirect – effector-mediated” indicates that the event enables downstream effector delivery or activity responsible for the immune outcome; “Indirect – injury-mediated” indicates that the immune outcome arises primarily from cellular stress, barrier disruption, membrane damage, or cell death; “Indirect – availability-mediated” indicates that regulation alters toxin abundance, dose, or timing rather than producing protein-level activation; and “Unresolved” indicates that a mechanistic connection between the specified event and immune remodeling has not been established.

For organizational clarity, Table 1 separates the toxin systems into three mechanistic layers: protein-level activation and processing; receptor-mediated uptake, intracellular trafficking, translocation, and vesicle-mediated deployment without demonstrated protein-level activation; and bacterial or phage-associated regulation of toxin expression and release. These layers may occur sequentially within the same intoxication pathway but are not mechanistically equivalent. The three downstream immune programs discussed below are recorded in the “Immune outcome” column and should not be interpreted as three categories of toxin activation.

## Host protease–mediated activation

Host protease–mediated cleavage is one of the most clearly defined mechanisms of bacterial toxin maturation [17,18]. By recognizing specific proteolytic motifs and removing inhibitory regions or separating functional domains, host proteases can generate toxin intermediates that are competent for subsequent oligomerization, translocation, or catalytic activity. However, the downstream host response should not automatically be attributed directly to the cleavage event itself, because proteolysis frequently functions as a prerequisite for productive intoxication rather than as the immediate cause of immune remodeling.

Furin, a calcium-dependent serine protease of the proprotein convertase family, is concentrated in the trans-Golgi network and endocytic compartments and recognizes polybasic cleavage motifs in several bacterial toxins [17]. Following endocytosis, *Pseudomonas aeruginosa* exotoxin A (PE) undergoes furin-mediated cleavage within domain II, generating a translocation-competent intermediate that ultimately delivers its ADP-ribosylating domain to the cytosol, where inhibition of host translation can secondarily compromise cellular defense functions [19]. Proaerolysin from *Aeromonas hydrophila* is processed by furin, PACE4, and PC5/6A in vitro. Cell-based experiments particularly support furin-mediated cleavage near the C terminus, which removes an inhibitory peptide and permits oligomerization and membrane pore formation [20]. The resulting membrane damage and inflammatory responses therefore occur downstream of toxin maturation rather than representing direct consequences of proteolysis.

Luminal proteases can also enhance toxin activity. In *Clostridium perfringens* enterotoxin (CPE), trypsin removes approximately 25 N-terminal residues and increases cytotoxic activity; the intact toxin is already active and should not be described as an obligatorily inactive protoxin. Structural studies suggest that this processing favors oligomerization, whereas claudin recognition is mediated by the C-terminal receptor-binding domain [21,22]. Subsequent pore formation damages epithelial membranes and increases mucosal permeability.

Diphtheria toxin (DT) further illustrates how proteolytic priming must be distinguished from subsequent intracellular deployment. Host furin cleaves intact DT into catalytic A and receptor-binding/translocation B fragments that remain connected by a disulfide bond, generating a primed A–B intermediate [23–25]. Following receptor-mediated endocytosis, progressive endosomal acidification drives conformational rearrangement and membrane insertion of the T domain, enabling translocation of the A fragment toward the cytosol. Low pH therefore promotes membrane insertion and translocation rather than directly activating the catalytic center. Once released into the cytosol, the A fragment ADP-ribosylates eEF-2 and rapidly arrests host protein synthesis [24]. Thus, proteolytic priming, intracellular translocation, catalytic action, and downstream cell injury represent sequential but mechanistically separable stages of DT intoxication.

## Host microenvironment–driven conformational remodeling and effector deployment

Host-derived physicochemical and metabolic cues can regulate toxin activation-associated processes by controlling conformational remodeling, membrane insertion, domain dissociation, intrinsic autoprocessing, and effector release. These processes frequently occur in defined intracellular compartments and thereby provide spatial control over productive intoxication. Importantly, however, not every host-triggered conformational change constitutes catalytic activation; in several toxin systems, environmental cues primarily regulate trafficking or translocation.

Endosomal acidification represents a major example. During extracellular transit, botulinum neurotoxins (BoNTs) can be incorporated into a progenitor toxin complex (PTC) in which non-toxic accessory proteins form a protective cage-like architecture that stabilizes the neurotoxin before release under appropriate physiological conditions [26,27]. Following receptor-mediated neuronal uptake, endosomal acidification drives conformational rearrangement of the heavy-chain translocation domain (HN), membrane insertion, and formation of a conduit through which the light chain reaches the cytosolic interface [27–31]. Reduction of the interchain disulfide bond by host redox machinery subsequently releases the light chain from the heavy chain [32]. These steps provide cytosolic access to the preformed Zn^2+^-dependent protease but do not directly switch on its catalytic center; enzymatic activity becomes manifest when the released light chain encounters its soluble NSF attachment protein receptor (SNARE) substrates. The resulting blockade of neurotransmitter release may subsequently produce tissue- and context-dependent neuroimmune effects [33,34].

DT uses a related low-pH-dependent translocation strategy. Following furin-mediated priming, endosomal acidification drives T-domain insertion and delivery of the catalytic A fragment into the cytosol rather than directly activating its enzymatic center [23,24]. *Mycoplasma pneumoniae* community-acquired respiratory distress syndrome (CARDS) toxin provides a further example in which acidification regulates intracellular deployment. CARDS toxin undergoes Annexin A2-mediated uptake and exploits endosomal acidification to promote conformational remodeling and KELED-dependent retrograde trafficking; it subsequently reaches intracellular compartments where perturbation of vacuolar ATPase-associated machinery contributes to vacuolization and epithelial injury [35,36]. In this context, acidification is best regarded as a spatial gate for productive toxin deployment rather than as a demonstrated catalytic activation event.

A reductive intracellular environment provides another important checkpoint for A–B toxins. In cholera toxin (CT), proteolytic nicking of the exposed loop connecting CTA1 and CTA2 generates two chains that remain linked by a disulfide bond. Bacterial proteases can produce nicked CT, and trypsin can process the unnicked A chain [37]. In polarized intestinal epithelial cells, apical entry also permits A-chain nicking by a host serine-type protease [38]. Following receptor-mediated uptake and retrograde trafficking through the Golgi apparatus to the endoplasmic reticulum (ER), reduction of the interchain disulfide bond and protein disulfide isomerase-assisted dissociation release CTA1 from the CTA2–B5 complex, while endoplasmic reticulum-associated degradation (ERAD) machinery facilitates CTA1 export toward the cytosol [2]. Once delivered to the cytosol, CTA1 interacts with host ADP-ribosylation factor (ARF) proteins, which stabilize a catalytically competent conformation and promote efficient ADP-ribosylation of Gsα [2,39,40]. The resulting cAMP elevation can subsequently alter dendritic-cell cytokine output, including reduced IL-12 production through impaired IRF8 function [39]. CT therefore exemplifies a multistep sequence in which proteolytic processing, ER dissociation, cytosolic delivery, ARF-dependent catalytic activation, and downstream immunological effects should be considered separately.

Shiga toxin (Stx) undergoes a related but distinct series of host-cell processing events. Following globotriaosylceramide (Gb3)-mediated uptake, Stx is transported retrogradely through endosomes and the Golgi apparatus to the ER [41–44]. During intracellular transport, host furin can cleave the protease-sensitive loop of the A chain at an Arg-X-X-Arg motif, generating A1 and A2 fragments that remain connected by the intraloop disulfide bond; trypsin can also nick this region [45,46]. ER reduction promotes release of the catalytic StxA1 fragment, which subsequently exploits an ERAD-like retrotranslocation pathway to enter the cytosol, where it depurinates 28S rRNA and inhibits protein synthesis [45–50]. Ribotoxic stress, epithelial and endothelial injury, and context-dependent modulation of inflammatory pathways occur downstream of this intracellular effector activity [51,52]. These protein-processing events must be distinguished from SOS-dependent prophage induction, which regulates Stx production and release rather than activating the toxin molecule itself.

Host-derived metabolites can also serve as highly specific triggers for intrinsic toxin autoprocessing. Certain toxins of the repeat-in-toxin (RTX) family contain self-processing modules in which Ca^2+^ binding stabilizes a cleavage-competent conformation and promotes Asp–Pro autoproteolysis, resulting in release of functional effector fragments [53,54]. A related but mechanistically distinct strategy is used by large clostridial glucosylating toxins. TcdA, TcdB, TcsL, and TcsH share a modular architecture in which receptor-mediated uptake and low-pH-dependent membrane translocation precede exposure of the cysteine protease domain (CPD) to the host cytosol. Cytosolic inositol hexakisphosphate (InsP_6_) then activates CPD autoprocessing and releases the N-terminal glucosyltransferase domain (GTD). The liberated GTD subsequently modifies small GTPases, with Rho- and Ras-family substrate preferences varying among toxins, producing barrier disruption, inflammatory responses, and cell-death phenotypes that vary according to toxin subtype, host species, and cell type [3,4,55–62]. Receptor binding and translocation therefore constitute deployment steps, whereas InsP_6_-dependent CPD autoprocessing represents the discrete protein-processing event.

Multifunctional-autoprocessing repeats-in-toxin (MARTX) toxins extend this metabolite-dependent activation logic. InsP_6_-dependent activation of the MARTX cysteine protease domain promotes intramolecular processing and release of embedded effector modules, including the Makes Caterpillars Floppy-like (MCF) domain. CPD-dependent effector liberation and the subsequent activation of individual effectors are distinct steps. For MCF, host ARF binding stimulates an additional autoprocessing event; processed MCF then cleaves the C-terminal tails of Rab GTPases and promotes their degradation [40,63–65]. Related *Photorhabdus* Mcf toxins broaden this ARF-dependent paradigm. Mechanistic evidence is strongest for Mcf1, whereas activation and downstream effector activities of Mcf2 remain less completely defined [66,67]. These examples illustrate how sequential host-metabolite sensing and host-factor recognition can establish multiple checkpoints between intracellular toxin delivery and effector activity.

Membrane composition and receptor engagement provide an additional level of host control over pore-forming toxin deployment. Cholesterol-dependent cytolysins bind cholesterol-rich host membranes, oligomerize into prepore assemblies, and undergo conformational rearrangements that generate transmembrane β-barrel pores [68]. Recent receptor-defined pore-forming toxins further illustrate that host recognition can be mechanistically distinct from subsequent pore formation. Epx recognizes MHC class I molecules as host receptors, thereby linking toxin targeting to an immune-associated surface protein; however, whether this receptor interaction directly contributes to immune remodeling beyond facilitating intoxication remains unresolved [69]. Their associated inflammatory effects are predominantly downstream consequences of membrane damage, ionic imbalance, and cell death. Similarly, *Staphylococcus aureus* α-hemolysin (Hla) engages the host metalloprotease ADAM10 before oligomerization and pore formation [70]. ADAM10-dependent perturbation of the E-cadherin axis contributes to epithelial junction disruption, whereas pore-associated membrane stress can promote inflammasome activation and regulated cell death. Thus, receptor engagement and pore formation should be interpreted as sequential components of intoxication rather than being collapsed into a single activation event.

A recent example extends receptor-dependent deployment to a non-pore-forming protease. White et al. identified claudin-4 as a receptor for *Bacteroides fragilis* toxin (BFT) and showed that receptor binding promotes BFT-mediated cleavage of cell-surface E-cadherin [71]. This receptor-guided proteolysis links toxin targeting to epithelial junction injury. Within the framework used here, it illustrates receptor-dependent substrate access rather than a demonstrated conversion of a latent toxin precursor into an active enzyme.

Together, these examples demonstrate that host microenvironmental cues can regulate several distinct molecular stages, ranging from genuine protein-level processing to membrane translocation and effector deployment. Their common feature is spatial restriction by host-associated conditions, but their mechanistic consequences differ substantially. Accordingly, the linkage between a given host cue and immune remodeling must be evaluated at the level of the specific processing, delivery, or effector step rather than inferred from the presence of a common environmental trigger.

## Expression and release regulation

Bacterial regulation of toxin expression and release represents a mechanistically distinct layer from protein-level activation. These processes control toxin abundance, release timing, local exposure dose, and spatial availability but do not necessarily convert an inactive toxin precursor into a functionally active molecule. Separating these population-level regulatory mechanisms from molecular activation is particularly important for phage-encoded toxins and vesicle-associated delivery systems.

SOS-dependent prophage induction provides a canonical example. In *S. aureus*, expression of the Panton–Valentine leukocidin (PVL) genes *lukS-PV* and *lukF-PV* is influenced by the phage life cycle, with DNA damage-induced prophage entry into the lytic program increasing phage copy number, toxin gene expression, and lysis-associated release [5,6]. The resulting increase in extracellular toxin availability can enhance neutrophil injury and inflammatory amplification, but the prophage switch itself does not constitute protein-level PVL activation. A related phage-associated regulatory logic applies to streptococcal pyrogenic exotoxin C (SpeC) in *Streptococcus pyogenes*, where prophage state influences toxin expression and extracellular availability [7].

DT illustrates a distinct environmental regulatory mechanism. The *tox* gene is carried by toxigenic corynephages but is transcriptionally controlled by the diphtheria toxin repressor DtxR. Under iron-replete conditions, Fe^2+^-activated DtxR represses *tox* transcription, whereas iron limitation relieves this repression and permits increased toxin production [72,73]. This bacterial regulatory step controls toxin abundance, while furin-mediated processing, receptor-mediated uptake, low-pH-dependent translocation, and cytosolic eEF-2 modification occur subsequently as mechanistically separate stages of intoxication [24].

Stx provides the clearest example of why expression and release regulation should not be labeled as protoxin activation. DNA damage, oxidative stress, or certain antibiotics activate the RecA-dependent SOS response, relieve repression of lambdoid prophage genes, and initiate the lytic program, thereby increasing *stx* transcription and toxin production [8]. Progression of the lytic cycle can subsequently promote bacterial lysis and pulse-like Stx release, increasing the local exposure dose [9]. These events regulate toxin abundance, release timing, and host exposure but do not activate Stx at the protein level. Productive host-cell intoxication instead requires Gb3-mediated uptake, retrograde trafficking, A-chain processing, disulfide reduction, StxA1 release, and ERAD-like retrotranslocation before cytosolic ribosomal depurination occurs.

Population-level specialization provides another form of regulated toxin deployment. In *Yersinia entomophaga*, production of the large YenTc toxin is restricted to a specialized toxin-producing subpopulation and is coupled to type 10 secretion system-dependent controlled cell lysis. This strategy allows toxin release to be coordinated at the bacterial-population level rather than through activation of individual toxin molecules [74].

Vesicle-mediated transport further expands the spatial regulation of toxin deployment. Enterotoxigenic *Escherichia coli* (ETEC) heat-labile toxin (LT) can be packaged within outer membrane vesicles (OMVs), which influence extracellular stability and delivery to intestinal epithelial cells. Cytolethal distending toxin (CDT) can likewise undergo vesicle-associated delivery followed by retrograde trafficking, with the CdtB effector ultimately reaching the nucleus through nuclear localization mechanisms. Typhoid toxin provides a related but distinct example in which host-derived vesicular carriers mediate toxin export from infected cells and subsequent uptake by recipient cells [75–79]. These vesicle-associated processes regulate toxin stability, localization, and intercellular delivery rather than directly converting an inactive toxin molecule into an active state.

Taken together, protein-level activation and expression/release regulation should be regarded as complementary but non-equivalent layers of toxin deployment. Proteolytic maturation, redox-dependent dissociation, intrinsic autoprocessing, and host-factor-dependent catalytic activation can directly alter the molecular state of a toxin, whereas phage induction, transcriptional regulation, bacterial lysis, and vesicle-mediated transport principally determine toxin abundance, timing, or spatial availability. As summarized in Table 1, the downstream relationship with immune remodeling is likewise heterogeneous: in some systems it is mediated through effector activity, in others through barrier damage, cellular stress, membrane injury, or cell death, and in several cases the causal connection remains unresolved. This distinction provides a more rigorous basis for comparing toxin systems without assuming that all processes required for productive intoxication constitute equivalent forms of protoxin activation.

## Immune-modulatory programs downstream of bacterial toxin effector action

Toxin activation is not itself an immune outcome; rather, molecular processing and productive delivery enable toxin effectors to reach their sites of action. Once deployed, these effectors can reshape host immunity across molecular, cellular, and tissue scales by altering signaling networks, damaging epithelial barriers, dismantling humoral defenses, and changing immune-cell survival. The strength and direction of these effects depend on toxin architecture, target-cell identity, tissue context, toxin dose, and timing [10,11].

These immunomodulatory outcomes are not monolithic. Instead, they can be organized into three partially overlapping programs: (i) disruption of inflammatory signaling and repatterning of cytokine networks; (ii) dismantling of antibodies, complement components, and chemotactic mediators; and (iii) induction of regulated cell-death programs that deplete immune or barrier-cell populations [12,13]. Figure 1 summarizes representative mechanisms within these programs while emphasizing that the resulting immune phenotypes may be direct consequences of toxin effector activity or secondary consequences of cellular and tissue injury.

**Figure 1.**
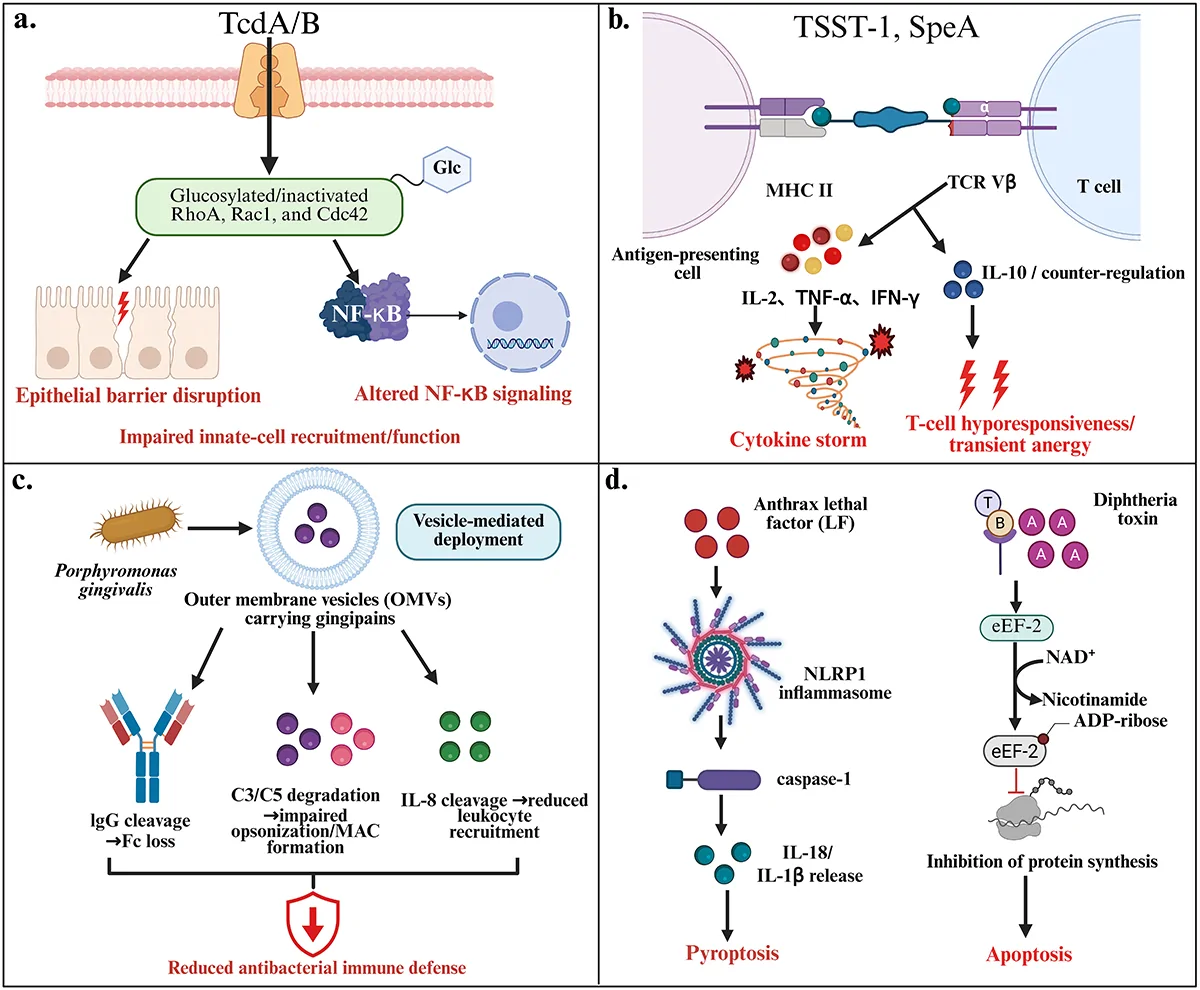
Representative immune-modulatory programs downstream of bacterial toxin effector action. (a) *Clostridioides difficile* toxins A and B (TcdA/B) glucosylate and inactivate RhoA, Rac1, and Cdc42, leading to epithelial barrier disruption, altered NF-κB signaling, and impaired innate immune-cell recruitment and function. (b) The superantigens toxic shock syndrome toxin-1 (TSST-1) and streptococcal pyrogenic exotoxin A (SpeA) bridge major histocompatibility complex class II and the T-cell receptor Vβ region, driving acute cytokine release; counter-regulatory responses during sustained or repeated exposure may subsequently promote T-cell hyporesponsiveness or transient anergy. (c) *Porphyromonas gingivalis* outer membrane vesicles (OMVs) deliver gingipains that cleave IgG, complement components, and IL-8, thereby weakening opsonization, membrane attack complex formation, and leukocyte recruitment. (d) In susceptible host contexts, anthrax lethal factor (LF) activates NLRP1–caspase-1 signaling and promotes IL-1β/IL-18 release and pyroptosis, whereas diphtheria toxin (DT) ADP-ribosylates eEF-2, inhibits protein synthesis, and can trigger apoptosis. These panels summarize downstream outcomes after productive intoxication and do not imply that toxin activation itself directly causes each immune phenotype. Created with BioRender.com.

## Disrupting inflammatory signaling and repatterning cytokine networks

Once toxin effectors reach their cellular sites of action, some preferentially intercept inflammatory signaling and cytokine networks rather than causing immediate cytolysis. By deploying a coupled strategy of signaling-hub disruption and network repatterning, they can create permissive windows for bacterial colonization and expansion. At the molecular level, hub disruption proceeds through distinct, often concerted, modalities: (i) enzymatic modification, in which effectors catalyze covalent modifications – including ADP-ribosylation, glucosylation, and deamidation – to lock signaling proteins into aberrant activity states [55,80,81]; (ii) molecular mimicry and competitive inhibition, whereby effectors occlude interaction surfaces or occupy adaptor hubs to prevent higher-order signalosome assembly [82]; and (iii) targeted degradation, in which ubiquitin- or proteasome-dependent elimination of regulatory components dismantles signaling competence [83]. These toxins frequently converge on essential nodes such as Rho-family GTPases, mitogen-activated protein kinase (MAPK) cascades, and TLR–NF-κB signaling axes, although the direction and magnitude of the resulting immune response remain context dependent [82].

TcdA/B from *Clostridioides difficile* (*C. difficile*) provide a prototypical example. Their glucosyltransferase domains glucosylate and inactivate Rho-family GTPases, including RhoA, Rac1, and Cdc42. This modification disrupts actin dynamics and epithelial junctional integrity while altering inflammatory signaling in a cell- and context-dependent manner. Cytoskeletal collapse can impair innate immune-cell migration and antimicrobial function, accompanied by epithelial barrier disruption and altered NF-κB output (Figure 1(a)). In selected myeloid-cell contexts, TcdB-mediated Rho glucosylation can activate the pyrin inflammasome and promote IL-1β/IL-18 release; this response should not be generalized to all biochemical mechanisms of Rho inactivation [57,84]. Thus, the same toxin-driven modification may amplify inflammatory tissue injury while weakening effective immune clearance [55–58].

MARTX toxins illustrate this mechanistic distinction. The Rho inactivation domain (RID) catalyzes lysine Nε-fatty acylation in the C-terminal polybasic region of Rho-family GTPases, a modification distinct from TcdB-mediated glucosylation [85]. RID-mediated Rho inactivation did not activate the pyrin inflammasome in the tested system [84]. In human intestinal epithelial cells, the *Vibrio cholerae* MARTX effectors RID and α/β hydrolase (ABH) suppress Rac1-dependent ERK, p38, and JNK activation, thereby limiting CXCL8, TNF, and other proinflammatory responses that would otherwise follow actin cross-linking domain-induced cytoskeletal injury [86]. Thus, Rho-family GTPase inactivation can silence inflammatory signaling without producing the inflammasome response associated with other toxin-induced modifications.

Immune effects also vary with the effector repertoire of *Vibrio vulnificus* MARTX toxins. The Ras/Rap1-specific endopeptidase (RRSP) cleaves the Switch I region of Ras and Rap1 and interrupts downstream ERK signaling [87]. The RID-dependent transforming NADase domain (RDTND), acting together with RID, depletes NAD(P)+ and modifies Rac1, suppressing reactive oxygen species (ROS) generation and associated ERK/JNK/NF-κB responses in phagocytes; this promotes bacterial dissemination in infection models [88]. These established effector-dependent effects should be distinguished from the still-unresolved specific immune consequences of MCF-mediated Rab processing [65]. Table 1 therefore separates MARTX CPD-dependent effector release from ARF-dependent MCF activation and records variant-dependent suppression of inflammatory signaling and ROS production.

A similar bidirectional pattern is evident in *S. aureus*. Its α-hemolysin (Hla) mediates a substantial component of its immunopathological activity through engagement of the host metalloprotease ADAM10, promoting ADAM10-dependent E-cadherin cleavage, epithelial junction disruption, and barrier dysfunction [70]. In parallel, Hla oligomerizes to form heptameric pores that induce K^+^ efflux and activate the NLRP3 inflammasome, contributing to IL-1β release and inflammatory amplification [89]. In contrast, phenol-soluble modulins (PSMα1–3) can dampen receptor-proximal signaling by antagonizing TLR4 and constraining NF-κB activation [90]. The coexistence of inflammatory escalation with parallel signal suppression can undermine coordinated host responses and bias immunity toward tissue injury rather than effective bacterial control.

Beyond canonical pore-forming toxins that primarily engage damage-associated inflammatory pathways, newly identified toxins indicate that receptor selection itself may shape immune interactions. *Enterococcus* pore-forming toxins (Epxs) provide a distinct example: Epx2 and Epx3 recognize major histocompatibility complex class I (MHC-I/HLA-I) molecules as host receptors, linking pore formation to a central component of immune surveillance. Interferon-mediated upregulation of MHC-I further increases cellular susceptibility to Epx toxicity, suggesting that immune-state-dependent receptor availability may influence toxin sensitivity. However, whether Epx directly remodels immune signaling beyond receptor-dependent cytotoxicity remains unresolved [69].

Beyond signaling-hub disruption, systemic repatterning of cytokine networks can further broaden immune imbalance. Superantigens such as toxic shock syndrome toxin-1 (TSST-1) and streptococcal pyrogenic exotoxin A (SpeA) simultaneously engage MHC class II molecules on antigen-presenting cells and the Vβ region of T-cell receptors on neighboring T cells, bypassing conventional peptide-specific recognition and activating large Vβ-defined fractions of the T-cell population. This response can drive abrupt release of IL-2, TNF-α, and IFN-γ and produce cytokine storm-like pathology (Figure 1(b)) [91,92]. Counter-regulatory IL-10 responses and sustained or repeated superantigen exposure may subsequently promote transient T-cell hyporesponsiveness or anergy [93].

By contrast, other toxins skew more directly toward immunosuppression by sustaining IL-10 elevation and establishing a tolerogenic milieu. In *Helicobacter pylori* (*H. pylori*) infection, VacA reprograms lamina propria macrophages and dendritic cells toward IL-10-dominant phenotypes, promotes peripheral Treg differentiation, and suppresses effector T-cell expansion, thereby facilitating persistence [94].

CT and adenylate cyclase toxin (CyaA) exemplify cAMP-centered reprogramming of dendritic-cell function. CT suppresses IL-12 production and alters dendritic-cell differentiation through cAMP-mediated inhibition of IRF8 function, thereby weakening Th1 responses [39]. Similarly, *Bordetella pertussis* CyaA rapidly raises intracellular cAMP, inhibits TLR-induced IRF-1/IRF-8 activation, suppresses IL-12p70, and enhances IL-10 through MAPK-linked reprogramming, yielding dendritic cells with reduced co-stimulatory capacity and more tolerogenic features [95].

## Dismantling humoral effector molecules

Some toxins directly compromise humoral immune components – including antibodies, complement, and chemotactic cues such as IL-8 – thereby weakening recognition, opsonization, and bactericidal amplification at the source [96]. During early acute infection, such “effector-dismantling” strategies can rapidly sever key defense links and produce highly efficient local immune evasion.

Humoral defense can also be weakened indirectly by injury to the phagocytes that execute antibody- and complement-dependent clearance. *Staphylococcus aureus* γ-hemolysins HlgAB and HlgCB target phagocytes through specific chemokine or complement receptors [97]. Their components induce divalent-cation influx followed by increased membrane permeability [98]. These cellular effects should be distinguished from the direct proteolytic destruction of antibodies or complement components discussed below.

In Gram-negative opportunists, secreted proteases can directly undermine complement. In *P. aeruginosa*, elastase (LasB) and alkaline protease (AprA) target key complement nodes. AprA blocks classical- and lectin-pathway activation primarily through cleavage of C2, leading to reduced C3b deposition and diminished C5a generation, whereas LasB is more broadly implicated in complement evasion at the review level. Together, these protease-mediated effects weaken opsonization, phagocyte recruitment, and complement-dependent killing, helping create a locally permissive humoral milieu that may contribute to persistence in chronic airway and biofilm-associated infection [99,100].

*Porphyromonas gingivalis* secretes the cysteine proteases RgpA/RgpB and Kgp and can deliver gingipain-enriched cargo through OMVs, extending proteolytic activity beyond the immediate bacterial surface. Gingipains can cleave IgG and impair antibody-dependent defense [101], degrade complement components including C3 and C5 and thereby disrupt complement-dependent opsonization and terminal effector activity [102,103], and modulate IL-8 activity [104] (Figure 1(c)). These effects depend on protease concentration and presentation: soluble gingipains can initially enhance IL-8 activity before degrading it, whereas vesicle-associated gingipains rapidly inactivate the chemokine; complement activation can likewise precede degradation under different protease conditions [103,104]. Thus, gingipains can combine local inflammatory stimulation with loss of effective humoral defense.

## Inducing programmed cell death to create immune depletion

Beyond signaling disruption and humoral-defense dismantling, toxin effector activity can impose a more radical form of immune weakening by inducing programmed death of immune or barrier cells, thereby depleting cellular effectors and creating functional gaps in host defense [14,15]. Toxin-associated death programs include apoptosis, pyroptosis, and necroptosis; despite their distinct upstream triggers, these pathways can converge on immune-cell loss, impaired antimicrobial coordination, and tissue injury [15,16].

DT exemplifies translation-arrest-associated cell death. Its catalytic A fragment transfers ADP-ribose from NAD^+^ to the diphthamide residue of eukaryotic elongation factor 2 (eEF-2), thereby inhibiting translation elongation. Sustained protein-synthesis arrest compromises cell survival [24]. In selected human epithelial models, eEF-2-inactivating toxins, including DT, can also engage ZAKα/p38-dependent NLRP1 inflammasome signaling and promote barrier injury [105]. By contrast, anthrax lethal factor can proteolytically cleave susceptible NLRP1 family sensors, leading to caspase-1 activation, IL-1β/IL-18 maturation and release, and pyroptotic death of susceptible macrophage-lineage cells (Figure 1(d)) [106,107].

Pore-forming toxins provide another route toward regulated membrane rupture. In *S. aureus* infection, Hla intoxication, involving both ADAM10-dependent signaling perturbation and pore-induced membrane stress, has been linked to activation of the RIPK1–RIPK3–MLKL axis in macrophages, driving necroptosis characterized by regulated membrane disruption and release of pro-inflammatory contents. This process contributes to lung tissue injury, impaired neutrophil-dependent defense, and immune-cell dysfunction during severe infection [108,109]. Finally, *H. pylori* VacA illustrates autophagy-associated injury: it induces LC3-II accumulation and autophagosome formation while impairing lysosomal acidification and autophagosome–lysosome fusion, thereby stalling autophagic flux [110]. Progressive autophagosome accumulation can cause cellular dysfunction, weaken local host defense, and bias immunity toward tolerance [94,110].

Although toxin effectors can reshape host immunity through disruption of inflammatory signaling, repatterning of cytokine networks, dismantling of humoral effectors, and depletion of immune cells, these outcomes should not be interpreted as mechanistically equivalent across toxin systems. In some contexts, immune remodeling is a direct consequence of intracellular effector action; in others, it arises secondarily from cytotoxicity, epithelial barrier disruption, or tissue injury. Moreover, similar toxin inputs can produce inflammatory amplification, immune hyporesponsiveness, or selective cell loss depending on host species, cell type, toxin dose, and the temporal stage examined. These considerations argue against treating post-deployment immune phenotypes as a single unified program and emphasize the need to distinguish direct immunomodulatory mechanisms from downstream pathological consequences.

## Case studies of immune modulation via toxin activation

### Anthrax toxin: furin-mediated priming, endosomal gating, and immune modulation

Anthrax toxin (AT) is the central virulence system of *Bacillus anthracis* (*B. anthracis*) and comprises protective antigen (PA), lethal factor (LF), and edema factor (EF). Rather than relying on a single activation event, AT follows a stepwise sequence involving PA proteolytic processing, receptor-mediated uptake and endosomal translocation, and cytosolic effector action. Cell-surface furin cleavage converts PA83 into oligomerization-competent PA63, whereas subsequent endosomal acidification drives pore formation and LF/EF translocation rather than directly activating their catalytic domains [111].

The cascade begins when PA83 binds the host receptors ANTXR1/2 (TEM8/CMG2), whose von Willebrand factor A domains and metal ion-dependent adhesion site (MIDAS) motifs support high-affinity toxin recognition [112]. Cell-surface furin then cleaves PA83 after the polybasic activation sequence, removing the approximately 20-kDa PA20 fragment and generating PA63 (Figure 2, step 1) [113]. PA63 assembles into heptameric or octameric prepore complexes and recruits LF and/or EF (Figure 2, step 2) [113]. Following LF/EF recruitment, the receptor-bound complex undergoes clathrin-mediated endocytosis and enters progressively acidifying endosomes (Figure 2, step 3) [114].

**Figure 2.**
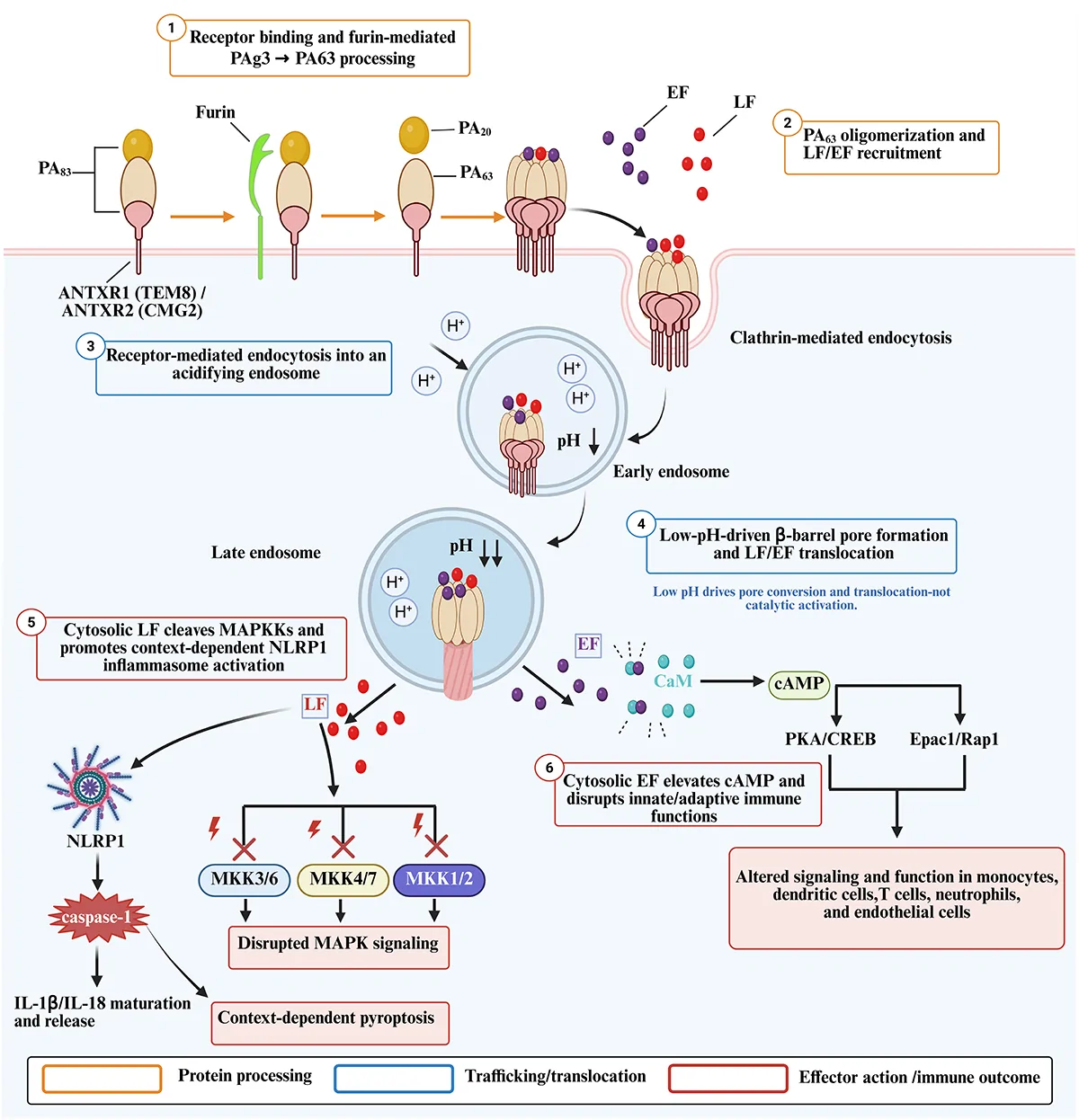
Stepwise processing, translocation, and context-dependent immune outcomes of anthrax toxin. The figure distinguishes protein processing (orange), trafficking/translocation (blue), and effector action and immune outcomes (red). (1) Protective antigen 83 (PA83) binds anthrax toxin receptors 1 and 2 (ANTXR1/2; TEM8/CMG2) and is cleaved by furin to generate oligomerization-competent PA63. (2) PA63 assembles into heptameric or octameric prepore complexes and recruits lethal factor (LF) and/or edema factor (EF). (3) The receptor-bound complex undergoes clathrin-mediated endocytosis and enters progressively acidifying endosomes. (4) Low pH drives prepore-to-pore conversion and formation of a transmembrane β-barrel channel, enabling LF/EF translocation rather than directly activating their catalytic domains. (5) Cytosolic LF cleaves mitogen-activated protein kinase kinases, disrupts MAPK signaling, and, in susceptible host contexts, promotes NLRP1 inflammasome activation, caspase-1-dependent IL-1β/IL-18 maturation and release, and pyroptosis. (6) Cytosolic EF elevates cAMP and alters innate and adaptive immune-cell functions through PKA/CREB- and Epac1/Rap1-associated signaling. Created with BioRender.com.

As endosomal pH decreases to approximately 5.0–6.0, PA domain 2 undergoes conformational remodeling, and its β-hairpins refold into a transmembrane β-barrel pore containing the φ-clamp (Figure 2, step 4) [114,115]. This low-pH transition functions as a trafficking and translocation checkpoint rather than as direct catalytic activation of LF or EF. Productive delivery requires partial unfolding and controlled passage of LF/EF through the PA channel before the effectors reach the cytosol.

In the cytosol, LF functions as a zinc-dependent metalloprotease that cleaves multiple MAPK kinases (MAPKKs), including MEK1/2, MKK3/6, and MKK4/7, thereby disrupting MAPK signaling. In specific host contexts, LF also promotes NLRP1 inflammasome activation, leading to caspase-1-dependent processing and release of IL-1β and IL-18 and context-dependent pyroptosis (Figure 2, step 5) [107]. In parallel, cytosolic EF acts as a Ca^2+^/calmodulin-dependent adenylate cyclase that elevates intracellular cAMP. Through the PKA/CREB and Epac1/Rap1 pathways, EF alters signaling and function in monocytes, dendritic cells, T cells, neutrophils, and endothelial cells (Figure 2, step 6) [116]. Thus, LF and EF generate distinct downstream immune effects after a shared PA-mediated entry and translocation pathway.

Structural biology has progressively established the molecular basis of this precision transport system: the PA monomer crystal structure was solved in 1997, providing an initial framework for mechanistic studies [117]. In 2004, structure-guided modeling proposed a prepore-to-pore transition and suggested endosomal acidification as the trigger [118]. In 2009, the field moved beyond a heptamer-only model by confirming the coexistence of functional heptameric and octameric oligomers [113]. A 2012 synthesis clarified how low pH drives PA conformational rearrangement and pore formation [114]. In the near-atomic era, the PA pore was resolved at atomic detail in 2015, elucidating the essential role of the φ-clamp in translocation [114,115]. Cryo-EM in 2020 further captured asymmetric LF-loaded heptameric prepore assemblies [119]. Despite these advances, how compartmentalized host environments quantitatively tune translocation kinetics – and how this “slow-release” logic shifts immune-recognition thresholds – remains a key frontier for integrative analysis.

### Botulinum neurotoxin: acidification-gated translocation, reduction-mediated light-chain release, and SNARE-targeted neuro-immune suppression

Botulinum neurotoxin (BoNT), produced by *Clostridium botulinum*, is among the most potent known A–B protein toxins. BoNT is synthesized as a single-chain precursor and proteolytically nicked to generate a heavy chain (HC) and a light chain (LC) that remain connected by an interchain disulfide bond [27]. During extracellular transit, BoNT can be incorporated into a PTC, in which nontoxic accessory proteins form a protective cage-like assembly that stabilizes the neurotoxin and permits its release under appropriate physiological conditions [26,27]. Following physiological dissociation of the PTC, free BoNT can access neuronal receptors (Figure 3, inset).

**Figure 3.**
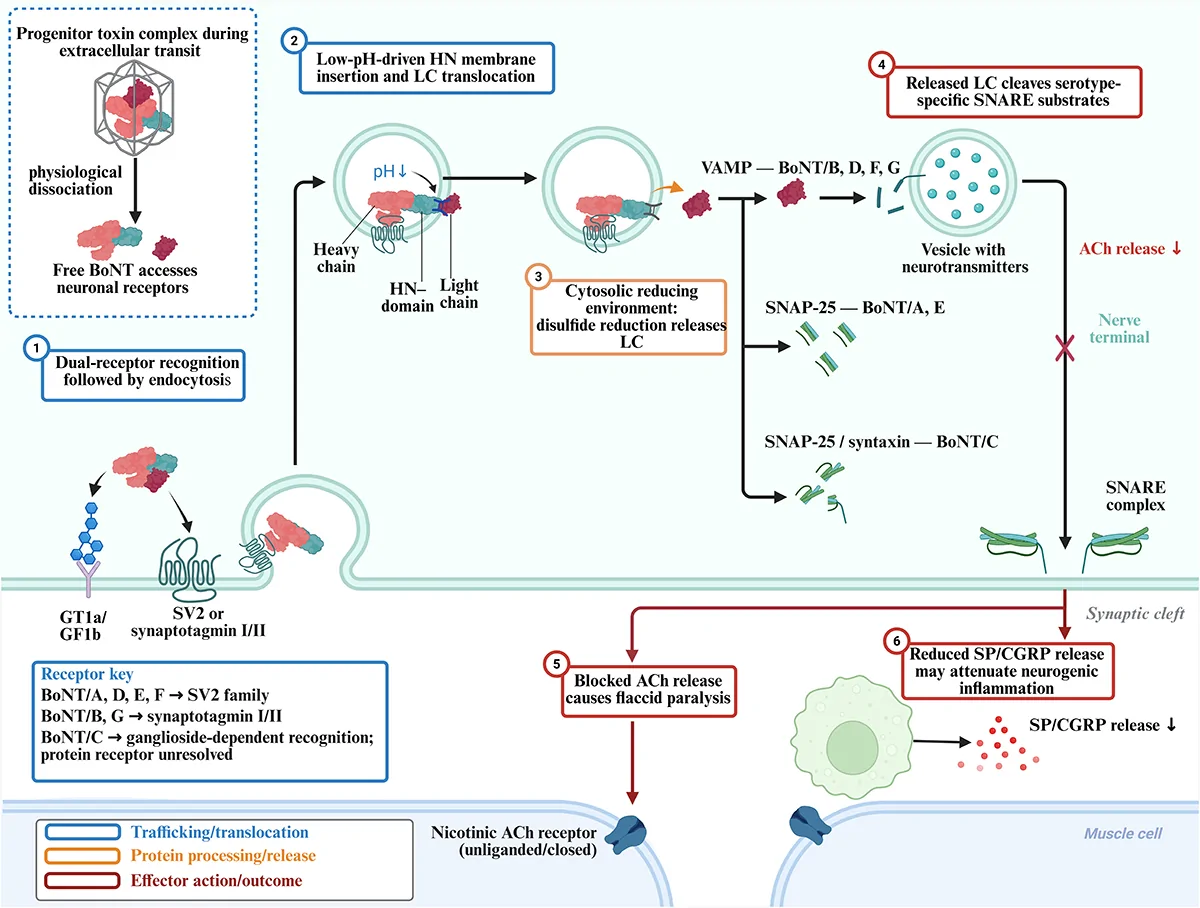
Acidification–reduction gating of botulinum neurotoxin translocation and SNARE targeting. The figure separates extracellular protection, neuronal uptake and translocation (blue), reduction-mediated protein release (orange), and effector action and outcomes (red). The inset depicts a representative progenitor toxin complex (PTC), in which nontoxic accessory proteins protect botulinum neurotoxin (BoNT) during extracellular transit and physiological dissociation releases free toxin for neuronal receptor engagement. The schematic subsequently depicts proteolytically nicked BoNT, whose heavy chain (HC) and light chain (LC) remain linked by an interchain disulfide bond. (1) Free BoNT recognizes neuronal gangliosides together with serotype-dependent protein receptors and undergoes endocytosis. Synaptic vesicle glycoprotein 2 family members serve as receptors for BoNT/A, BoNT/D, BoNT/E, and BoNT/F; synaptotagmin I/II serve as receptors for BoNT/B and BoNT/G; and BoNT/C exhibits ganglioside-dependent recognition, with its protein receptor remaining unresolved. (2) Endosomal acidification drives insertion of the heavy-chain translocation domain and LC translocation rather than directly activating LC catalysis. (3) At the cytosolic interface, the thioredoxin/thioredoxin reductase system reduces the interchain disulfide bond and releases LC from HC. (4) Released LC cleaves serotype-specific soluble N-ethylmaleimide-sensitive factor attachment protein receptor (SNARE) substrates: vesicle-associated membrane protein for BoNT/B, BoNT/D, BoNT/F, and BoNT/G; SNAP-25 for BoNT/A and BoNT/E; and SNAP-25 plus syntaxin for BoNT/C. (5) In motor terminals, inhibition of acetylcholine release causes flaccid paralysis. (6) In sensory terminals, reduced substance P and calcitonin gene-related peptide release may attenuate neurogenic inflammation; this is an indirect, context-dependent effect and should not be generalized to all BoNT serotypes. Created with BioRender.com.

Entry begins with dual-receptor recognition: the C-terminal receptor-binding domain of HC engages neuronal gangliosides such as GT1a and GT1b together with serotype-dependent synaptic vesicle protein receptors. As summarized in Figure 3, synaptic vesicle glycoprotein 2 (SV2) family members serve as receptors for BoNT/A, BoNT/D, BoNT/E, and BoNT/F, whereas synaptotagmin I/II serve as receptors for BoNT/B and BoNT/G. For BoNT/C, ganglioside-dependent recognition is established, but its protein receptor remains unresolved (Figure 3, step 1) [27,30]. Receptor engagement is followed by uptake into recycling synaptic vesicles.

As luminal pH decreases, the heavy-chain N-terminal translocation domain (HN) undergoes conformational rearrangement, exposing hydrophobic helices that insert into the endosomal membrane and form an ion-conductive translocation channel through which LC reaches the cytosol (Figure 3, step 2) [27,31]. Low pH therefore licenses membrane insertion and LC translocation rather than directly activating the LC catalytic center. A subsequent release checkpoint occurs at the cytosol-facing interface: the thioredoxin/thioredoxin reductase system reduces the interchain disulfide bond linking LC and HC, allowing LC release into the cytoplasm (Figure 3, step 3) [32].

This sequential safeguard – acidification-driven translocation followed by reduction-mediated release – ensures that LC reaches the cytosol as a freely diffusible enzyme. Once released, LC cleaves its cognate SNARE substrates rather than undergoing direct catalytic activation by low pH. The serotype-specific targets include vesicle-associated membrane protein (VAMP) for BoNT/B, BoNT/D, BoNT/F, and BoNT/G; synaptosomal-associated protein of 25 kDa (SNAP-25) for BoNT/A and BoNT/E; and both SNAP-25 and syntaxin for BoNT/C (Figure 3, step 4) [27]. SNARE cleavage prevents productive ternary-complex assembly and synaptic vesicle fusion, thereby blocking acetylcholine release and causing flaccid paralysis (Figure 3, step 5) [27].

Beyond neuromuscular blockade, BoNT/A can indirectly modulate inflammation through the neuroimmune axis. In sensory terminals, BoNT/A-associated inhibition of substance P and calcitonin gene-related peptide (CGRP) release may attenuate neurogenic inflammatory input (Figure 3, step 6) [33]. This outcome is indirect and context dependent and should not be generalized to all BoNT serotypes or attributed directly to the low-pH translocation or disulfide-reduction steps. Together with other anti-inflammatory effects reported in pain models [33,34], these observations support indirect neuroimmune modulation after successful LC delivery.

Structural work supports this compartmentalized deployment model. X-ray crystallography resolved the BoNT/A LC catalytic center, revealing the characteristic HEXXH zinc-binding motif and substrate-binding features [27]. Subsequent structural studies demonstrated low-pH-driven relaxation of the HN domain consistent with a translocation-competent intermediate [31]. Recent high-resolution cryo-EM further captured BoNT/A1–SV2B receptor complexes across different pH states, delineating transitions from semi-closed to open and ultimately translocation-adapted conformations driven by receptor engagement and acidification [30].

### Clostridioides difficile *toxin B: InsP_6_-triggered autoprocessing and context-dependent inflammatory remodeling*

TcdB is a principal driver of *C. difficile* pathogenicity. It exemplifies a host-metabolite-sensing activation mechanism mediated by its cysteine protease domain (CPD), which specifically recognizes the host cytosolic metabolite inositol hexakisphosphate (InsP_6_). InsP_6_ binding induces allosteric reconfiguration of the CPD and triggers autoprocessing, releasing the N-terminal glucosyltransferase domain (GTD) from the toxin scaffold and enabling autonomous catalytic action [3,4].

TcdB entry is initiated by receptor engagement through receptor-specific binding interfaces, rather than by the catalytic GTD. CSPG4 recognizes a composite interface involving the CPD, delivery and receptor-binding domain (DRBD), intervening hinge, and part of the CROPs, whereas FZD1/2/7 recognition involves a distinct interface within the DRBD (Figure 4, step 1) [120,121]. More recently, tissue factor pathway inhibitor (TFPI) was identified as a receptor for TcdB4 and certain related TcdB variants, revealing subtype-dependent switching between FZD- and TFPI-binding specificities [122]. Receptor engagement promotes receptor-mediated endocytosis (Figure 4, step 2). Within acidified endosomes, the translocation machinery undergoes hydrophobic rearrangement and inserts into the endosomal membrane, enabling translocation of the N-terminal toxin portion toward the cytosolic interface (Figure 4, step 3) [3,4]. InsP_6_-dependent CPD protease activation therefore follows cytosolic exposure; this catalytic role is distinct from the CPD contribution to the CSPG4-binding interface.

**Figure 4.**
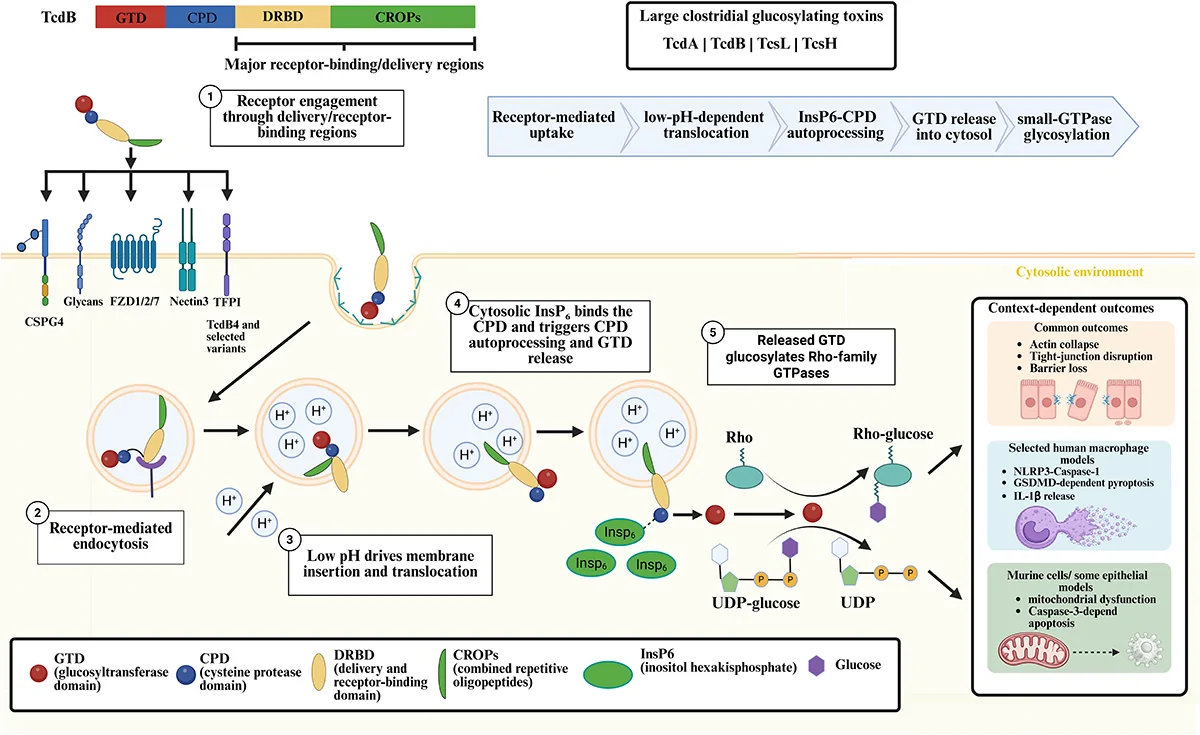
Receptor-dependent uptake, InsP_6_–CPD processing, and context-dependent outcomes of large clostridial glucosylating toxins. The figure uses TcdB as the representative mechanistic example; receptor usage and small-GTPase substrate preferences differ among toxins and TcdB variants. (1) TcdB engages receptor-specific binding interfaces rather than using the catalytic glucosyltransferase domain (GTD) for receptor recognition. Receptor contacts are schematic: CSPG4 binding involves a composite interface spanning CPD, DRBD, the hinge region, and part of the CROPs [120]. FZD1/2/7 and TFPI recognize distinct toxin variants through the DRBD; TFPI is used by TcdB4 and selected related variants [121,122]. Nectin-3 and glycans are included as reported receptor or attachment interactions, not as evidence of an identical binding site or contribution to uptake in every model. (2) Receptor engagement promotes endocytosis; the black connector denotes continuity of the toxin polypeptide, not a resolved linker conformation. (3) Endosomal acidification promotes membrane insertion and translocation rather than directly activating GTD catalysis. (4) Cytosolic InsP_6_ activates CPD autoprocessing and GTD release. (5) GTD glucosylates Rho-family GTPases, leading to cytoskeletal and barrier injury. The outcome panels show representative, non-exclusive cellular responses rather than a strict species-based division. In selected human macrophage models, NLRP3 activation and IL-1β release can occur independently of GTD activity; Pyrin responses and apoptosis depend on cell type and experimental context [57,62,84]. Created with BioRender.com.

At the cytosolic interface, InsP_6_ binds the CPD allosteric pocket and initiates active-site reorganization and autoprocessing, releasing the GTD into the cytosol (Figure 4, step 4) [3,4]. This mechanism, first systematically defined for TcdA, is conserved in TcdB. The released GTD subsequently glucosylates Rho-family GTPases, including RhoA, Rac1, and Cdc42, locking them in inactive states and driving actin collapse, tight-junction disruption, and loss of epithelial barrier integrity (Figure 4, step 5) [59].

Beyond barrier failure, TcdB can induce distinct inflammasome responses according to cell identity and priming state. In selected human monocyte-derived macrophage and macrophage-like cell models, TcdB activates NLRP3–caspase-1 signaling and IL-1β release without requiring GTD catalytic activity or Rho glucosylation [57,61]. In contrast, human monocytes and murine macrophages can respond through Pyrin, and prolonged inflammatory priming can license Pyrin responses in human macrophages [57,84]. Inflammasome signaling can promote gasdermin-mediated pyroptosis, but cytokine release should not be equated with uniform or extensive cell lysis [15,57]. TcdB also induces caspase-dependent and caspase-independent apoptosis in selected epithelial cell models [62]. The outcomes illustrated alongside Figure 4, step 5, are therefore representative and non-exclusive; they do not define a universal human-pyroptosis vs murine-apoptosis division or a single causal route downstream of Rho modification.

Although TcdB is discussed here in the greatest mechanistic detail, TcdA, TcsL, and TcsH share the modular organization and general intoxication sequence characteristic of large clostridial glucosylating toxins, including receptor-mediated uptake, low-pH-dependent membrane translocation, InsP_6_-triggered CPD autoprocessing, and GTD-mediated glycosylation of host small GTPases. However, these toxins differ in their receptor preferences, cellular tropism, glycosyl-donor usage, and GTPase substrate spectra. Figure 4 therefore presents TcdB as the detailed mechanistic example while placing TcdA, TcsL, and TcsH within the broader conserved large clostridial glucosylating toxin framework [3,4,59,123].

Structural studies have mapped several key mechanistic nodes. Early CPD–InsP_6_ crystal structures defined the ligand-binding pocket and catalytic geometry, whereas subsequent full-length TcdB structures revealed principles of multidomain organization [3,4]. Recent cryo-EM work further indicates that combined repetitive oligopeptides (CROPs) conformational dynamics influence FZD7 engagement and that the clinical antibody bezlotoxumab neutralizes TcdB by restricting CROPs conformations and occluding receptor interfaces [121]. Nevertheless, the complete dynamic sequence of InsP_6_-triggered CPD cleavage, as well as the mechanisms that stabilize the released GTD and enforce its substrate selectivity in the cytosol, remain incompletely resolved.

### Shiga toxin (Stx): phage-regulated expression and release, intracellular processing, and ribotoxic host injury

Stx is produced by *Shigella dysenteriae* type 1 and Shiga toxin–producing *E. coli* (STEC), including the O157:H7 lineage. In STEC, stx genes are commonly carried by lambdoid prophages. DNA damage, oxidative stress, or certain antibiotics activate the bacterial RecA-dependent SOS response, relieve repression by the phage CI repressor, and initiate lytic-cycle gene expression, thereby increasing *stx* transcription and toxin production (Figure 5, step 1) [8]. Progression of the prophage lytic program can subsequently promote bacterial lysis and lysis-associated Stx release, increasing the local exposure dose in a pulse-like manner (Figure 5, step 2) [9]. This bacterial regulatory process determines toxin abundance, release timing, and local exposure dose but does not activate the Stx molecule at the protein level.

**Figure 5.**
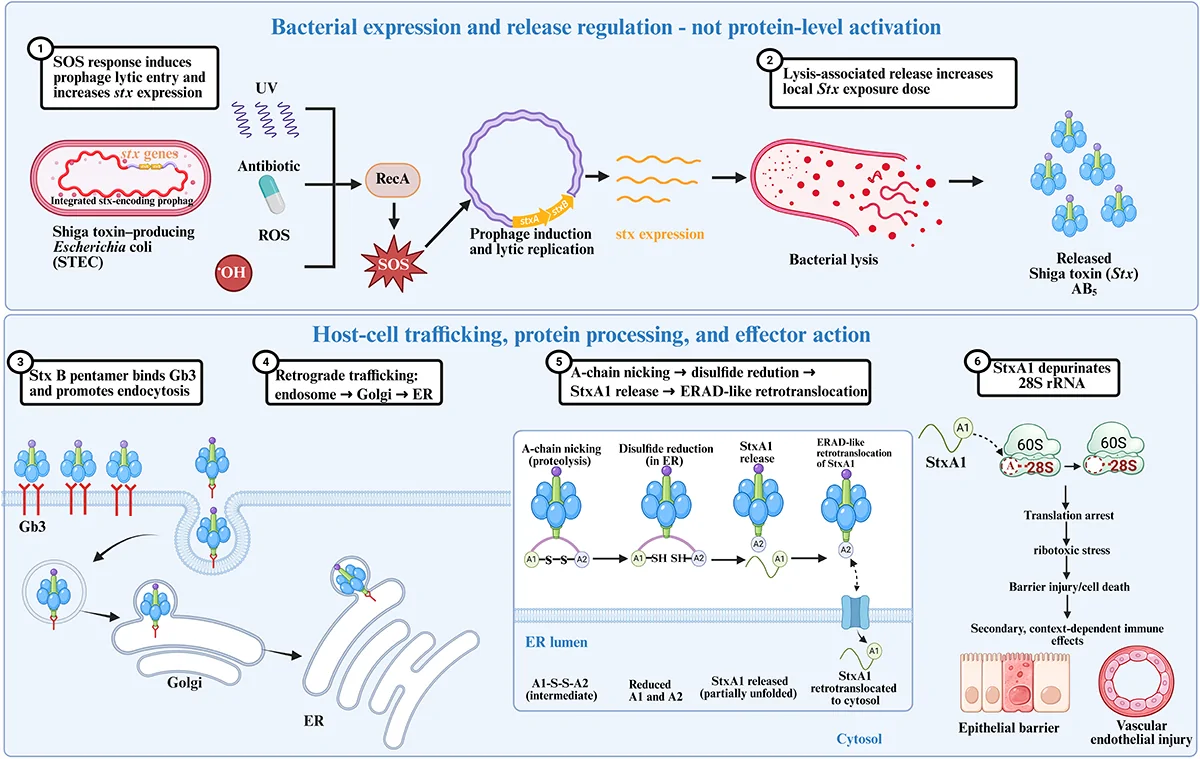
SOS-regulated Shiga toxin availability, intracellular processing, and ribotoxic host injury. The figure separates prophage-associated regulation of toxin production and release in STEC from host-cell trafficking, protein processing, and effector action. (1) DNA damage, oxidative stress, or certain antibiotics activate the RecA-dependent SOS response, promoting prophage induction, lytic-cycle gene expression, and increased *stx* expression; this regulates toxin abundance rather than activating the Shiga toxin (Stx) protein itself. (2) Progression of the lytic program promotes bacterial lysis and lysis-associated Stx release, increasing local toxin exposure. (3) The Stx B pentamer binds globotriaosylceramide (Gb3) and promotes host-cell internalization. (4) Internalized Stx undergoes retrograde trafficking from endosomes through the Golgi apparatus to the endoplasmic reticulum (ER). (5) Proteolytic nicking of the A chain generates disulfide-linked A1 and A2 fragments; in the ER, disulfide reduction promotes StxA1 release, followed by ER-associated-degradation-like retrotranslocation of StxA1 into the cytosol. (6) Cytosolic StxA1 depurinates 28S rRNA, resulting in translational arrest and ribotoxic stress. Subsequent epithelial and vascular endothelial barrier injury, cell death, and immune effects are secondary, context-dependent consequences of toxin-induced cellular injury. Created with BioRender.com.

Following host-cell uptake, Stx undergoes retrograde trafficking and sequential protein processing before its catalytic fragment reaches the cytosol. The Stx B pentamer binds the glycosphingolipid receptor Gb3 and promotes internalization (Figure 5, step 3), after which the toxin is transported retrogradely from endosomes through the Golgi apparatus to the endoplasmic reticulum (ER) (Figure 5, step 4) [44]. During intracellular transport, the Stx A chain can be proteolytically nicked, commonly by furin, into A1 and A2 fragments that remain connected by a disulfide bond [45,46]. In the ER, reduction of this disulfide bond promotes release of the catalytic StxA1 fragment from the A2–B5 complex [47]. StxA1 subsequently exploits an ERAD-like retrotranslocation pathway to reach the cytosol (Figure 5, step 5), where it depurinates 28S rRNA and inhibits protein synthesis (Figure 5, step 6) [48–50]. This ribotoxic lesion halts protein synthesis and induces cellular stress and cell death, compromising intestinal epithelial and vascular endothelial barriers and contributing to injury in high-Gb3 tissues such as the renal microvasculature [51]. Importantly, the downstream consequences of Stx intoxication extend beyond direct cytotoxicity. Ribotoxic stress, translational arrest, barrier disruption, and cell death can secondarily reshape inflammatory responses in a context-dependent manner. For example, Stx has been reported to interfere with non-canonical inflammasome responses and attenuate inflammasome-dependent cytokine output, including IL-1β, in specific cellular settings [52]. Thus, the immune consequences associated with Stx should be interpreted in the context of cell type, tissue compartment, and the extent of toxin-induced injury rather than as a direct consequence of SOS-mediated toxin release or protein processing itself.

Structural studies have progressively resolved key nodes in Stx receptor recognition and intracellular action. Crystallographic analyses established the Gb3-binding architecture and subtype differences of the B pentamer, while more recent structural studies of Stx and neutralizing antibody complexes have provided templates for therapeutic strategies targeting receptor engagement and toxin trafficking [124,125]. Yet, a comprehensive spatiotemporal model will need to integrate bacterial-level regulation of Stx expression and release with host-cell retrograde trafficking, proteolytic nicking of the A chain, ER disulfide reduction, StxA1 release, ERAD-like retrotranslocation, and the downstream consequences of ribotoxic stress.

## Conclusions and future perspectives

Comparison across representative toxin systems indicates that protein-level activation, intracellular deployment, and regulation of toxin expression or release operate at distinct mechanistic levels, even when they occur sequentially. Organizing the evidence according to these levels helps distinguish whether functional competence is established through molecular processing, whether productive intoxication depends primarily on trafficking or translocation, or whether host exposure is governed by changes in toxin expression and release. This separation provides a clearer basis for evaluating how individual steps relate to downstream immune remodeling without treating the entire intoxication pathway as a single activation continuum.

The relationship between these mechanistic processes and immune remodeling is not uniform across toxin systems. In some cases, a defined processing event is closely coupled to effector release and downstream host responses; in others, immune phenotypes arise primarily from subsequent effector activity, cytotoxicity, barrier disruption, regulated cell death, or altered toxin availability. Anthrax toxin, for example, undergoes receptor binding and furin-dependent PA processing before endosomal pore formation and LF/EF translocation [126], whereas TcdB requires cytosolic InsP_6_-dependent activation of its cysteine protease domain to release the glucosyltransferase domain for Rho-family GTPase modification [3,4]. By contrast, botulinum neurotoxin uses low-pH-triggered HN membrane insertion to translocate the light chain, followed by reduction-mediated light-chain release at the cytosolic interface; these delivery steps position the preformed catalytic domain to cleave SNARE substrates rather than directly activating its catalytic center [27]. Likewise, SOS-dependent prophage induction in Shiga toxin–producing bacteria regulates Stx abundance, release timing, and local exposure dose rather than activating the toxin protein itself, whereas subsequent host-cell trafficking, proteolytic A-chain nicking, ER disulfide reduction, StxA1 release, and ERAD-like retrotranslocation determine productive intracellular intoxication [8,9,44–49]. These comparisons emphasize that activation, deployment, and effector action should be resolved as related but non-equivalent mechanistic layers.

A second unresolved issue is how these molecular events translate into immune phenotypes. The evidence reviewed here indicates that toxin-associated immune remodeling may arise either from direct targeting of immune signaling machinery or indirectly through cytotoxicity, epithelial or endothelial barrier disruption, cellular stress, and regulated cell death. Toxin-mediated immune effects should therefore not be interpreted as an inevitable consequence of the activation step itself. For example, pore-forming toxins from *S. aureus* can promote inflammatory signaling and host-cell death in phagocytic populations [127], while *P. gingivalis* gingipains, whether released directly or delivered through outer membrane vesicles, can compromise antibody-, complement-, and chemokine-dependent defense mechanisms [101–104]. Similarly, the ribotoxic effects of Stx can lead to epithelial and vascular injury with secondary, context-dependent consequences for inflammatory signaling. Future studies will need to determine which immune phenotypes are directly coupled to toxin molecular processing and which instead emerge from downstream tissue injury or loss of immune-cell function.

Several priorities follow from these distinctions. First, future studies should experimentally separate molecular activation from intracellular trafficking, translocation, expression control, and release regulation rather than treating them as a continuous activation pathway. Second, activation site, translocation route, effector site, and immune outcome should be measured within the same experimental framework whenever possible. Third, the strength of evidence linking a specific activation event to a specific immune phenotype should be evaluated explicitly across toxin systems and host contexts. Fourth, tissue- and cell-type dependence should be addressed using physiologically relevant models, because identical toxin inputs may produce divergent inflammatory, cytotoxic, or immunosuppressive outcomes in different host compartments. Finally, therapeutic strategies should be matched to the mechanistic level being targeted: receptor engagement or intracellular delivery may be blocked independently of protein-level activation, whereas defined activation checkpoints such as InsP_6_-dependent TcdB autoprocessing may provide distinct opportunities for intervention. Structural and functional studies of TcdB have already identified receptor engagement, translocation, and InsP_6_-triggered autoproteolysis as separable molecular targets [3,4,122,123,128,129], while engineered Stx B-subunit immunogens and neutralizing antibodies aim primarily to prevent toxin entry and subsequent ribotoxic injury [125].

High-resolution structural approaches, organoid and tissue-based infection models, spatial omics, single-cell immune profiling, and intravital imaging will be particularly valuable when used to resolve these mechanistic layers rather than simply catalog downstream toxin effects. The next step for the field is therefore not to assume a universal continuum from protoxin activation to immune remodeling, but to determine, for each toxin system, where molecular activation occurs, how the active effector is subsequently deployed, and whether the resulting immune phenotype is a direct mechanistic consequence or a secondary outcome of intoxication. Such a context-resolved framework should clarify when protoxin activation represents a genuine regulatory checkpoint for host immune modulation and when it functions principally as one prerequisite within a broader intoxication pathway.

## Data Availability

Data sharing is not applicable to this article because no datasets were generated or analyzed during the current study.
